# Novel Biodegradable Starch Film for Food Packaging with Antimicrobial Chicory Root Extract and Phytic Acid as a Cross-Linking Agent

**DOI:** 10.3390/foods9111696

**Published:** 2020-11-19

**Authors:** Andrzej Jaśkiewicz, Grażyna Budryn, Agnieszka Nowak, Magdalena Efenberger-Szmechtyk

**Affiliations:** 1 Faculty of Biotechnology and Food Sciences, Institute of Food Technology and Analysis, Lodz University of Technology, Stefanowskiego 4/10, 90-924 Lodz, Poland; grazyna.budryn@p.lodz.pl; 2 Faculty of Biotechnology and Food Sciences, Institute of Fermentation Technology and Microbiology, Lodz University of Technology, Wólczańska 171/173, 90-924 Lodz, Poland; agnieszka.nowak@p.lodz.pl (A.N.); magdalena.efenberger-szmechtyk@edu.p.lodz.pl (M.E.-S.)

**Keywords:** chicory root, sesquiterpene lactones, antimicrobial activity, biodegradable film, starch, phytic acid

## Abstract

The aim of the study was to obtain and evaluate the properties of biodegradable starch film with the addition of phytic acid (0.05%) as a cross-linking agent and chicory root extract (1–5%) as an antimicrobial agent. To prepare biodegradable film, extracts from chicory root obtained with water or methanol were used. The content of bioactive compounds (sesquiterpene lactones and total polyphenols) was evaluated in chicory extracts. The antibacterial activity of the extracts was tested against Gram-negative bacteria (*Pseudomonas fluorescens*, *Escherichia coli*) and Gram-positive bacteria (*Bacillus subtilis*, *Staphylococcus aureus*) using the microculture method. The extracts acted as bacteriostatic agents, decreasing the growth rate (*µ_max_*), and extending the lag phase (*t_lag_*). The most sensitive bacterium in terms of film bacteriostatic activity was *P. fluorescens*; all extracts, irrespective of the solvent used, decreased its *µ_max_* value. *S. aureus* was the least sensitive. The obtained films were tested for their properties as food packaging (color, thickness, permeability, mechanical strength). Phytic acid improved the tensile strength and barrier properties of the films. The antimicrobial activity of the films was studied by the disk diffusion method against Gram-negative (*P. fluorescens*, *E. coli*) and Gram-positive (*B. subtilis*, *S. aureus*) bacteria, as well as fungi (*Candida albicans*, *Aspergillus niger*). The growth-inhibiting activity of each obtained film was observed for all tested microorganisms, and the most beneficial effect was observed for films with the 5% level of added extracts obtained with water. The growth-inhibiting activity for fungi, in particular for the yeast *C. albicans*, was low.

## 1. Introduction

Starch is the most important storage polysaccharide in plants, and it can be found in leaves, fruits, seeds, roots, tubers, stem cores, and rhizomes in the form of grains [1]. Physical, chemical, enzymatic, and genetic treatments of starch can induce novel gelatinizing, pasting, and retrogradation properties, and can improve the processing quality. An example of modifying starch properties is the use of a phytic acid additive, which acts as a starch phosphorescent agent, influencing the viscosity of the starch sol and gel [2]. Studies have also included starch films, mostly focused on reducing their flammability or improving their plasticity [3].

Phytic acid (myo-inositol hexakisphosphate) is a substance of natural origin obtained from cereals, oil seeds, and legume seeds [4]. It has attracted much attention in recent years as a food additive as an antibacterial agent and as a coating to make metals corrosion-resistant, because of its unique properties such as nontoxicity, low cost, good biocompatibility, and biodegradability [5]. Phytic acid occurs mainly in the aleuronic layer of small grains and is therefore found in high concentrations, for example, in wheat germ [6]. Since 2012, phytic acid has been approved as a Generally Recognized as Safe (GRAS) substance used as an antioxidant, chelating, and antibacterial agent in beverages and beverage bases, dairy products, processed vegetables, and vegetable juices [7]. Phytic acid was applied for modification of polylactic acid (PLA), improving its barrier properties [8].

Common chicory (*Cichorium intybus* L.) is a vegetable belonging to the *Asteraceae* family that is rich in bioactive compounds that affect human health. The leaves and flowers are usually used in salads [9]. Chicory roots are used on an industrial scale to obtain inulin as an ingredient of a coffee substitute after roasting and as animal feed [9,10,11]. Extracts from chicory are also used as additives in alcoholic and nonalcoholic beverages [12]. Due to its proven properties of stimulating and supporting the appetite and its prebiotic activity, *Cichorium intybus* extract is a component of dietary supplements and food for particular nutritional uses [13,14]. Besides inulin, there are valuable sesquiterpene lactones in chicory, mainly lactucine and lactucopicrin [15]. These lactones, apart from having a positive effect on human health, exhibit antimicrobial properties. The literature describes the antimicrobial effect of chicory extracts on Gram-positive and Gram-negative bacteria, as well as on fungi [16,17]. Chicory root extract has also been used in dairy products and inhibited the growth of *B. cereus* [18]. Moreover, chicory extract has been used as an additive in silk, not only as a dye, but for its antioxidant and antimicrobial properties against *S. aureus* and *E. coli*. [19].

The addition of various plant extracts to packaging films, besides improving their mechanical and barrier properties, may positively affect the antioxidant as well as antimicrobial activity [20,21]. Most often herbal, green tea, or spice extracts are used for this purpose [22,23]. Other examples of plant extracts used in biodegradable films are apple, blueberry, and rowan extracts. The available literature does not provide information on the use of chicory extracts in packaging films [24]. Due to its antimicrobial properties, chicory root extract can be applied in food packaging materials and may extend the shelf life of food products [19].

The purpose of the study was to obtain and evaluate the properties of starch biodegradable film with the addition of phytic acid as a cross-linking agent and chicory root extract as an antimicrobial agent. Physicochemical analyses of the designed starch films with the addition of phytic acid and chicory extract included thickness, water content, swelling, mechanical strength, and color measurements. The composition of the analyzed extracts was also examined, including the content of sesquiterpene lactones and total polyphenols. Microbiological tests included evaluating the inhibitory effect of the extracts themselves and the obtained biodegradable films.

## 2. Materials and Methods

### 2.1. Chemicals and Reagents

Potato starch was purchased from PEPEES (Łomża, Poland); glycerol ≥ 99.5% from Stanlab (Lublin, Poland); phytic acid 50% water solution, LC-MS grade solvents (water ≥ 95%, methanol ≥ 99.9%, formic acid ≥ 98%), and chlorogenic acid ≥ 95% from Sigma-Aldrich (St. Louis, MO, USA); methanol analytical grade ≥ 99.8%, concentrated hydrochloric acid 37%, and Fehling, Carrez, and Folin-Ciocalteu reagents from Chempur (Piekary Śląskie, Poland); calcium chloride anhydrous ≥ 97% and natrium carbonate anhydrous ≥ 98% from Eurochem (Tarnów, Poland); lactucin ≥ 95% and lactucopicrin ≥9 5% from Extrasynthese (Genay, France); and Tryptic Soy Broth (TSB), Tryptic Soy Agar (TSA), Sabouraud, and Malt Extract Agar (MEA) media from Merck (Darmstadt, Germany).

### 2.2. Microorganism Strains

The research material comprised microorganisms belonging to different taxonomic groups (bacteria, yeasts, molds) and characterized by different growth physiology and sensitivity to antimicrobial agents (Table 1). Microorganisms were obtained from American Type Culture Collection (ATCC) and National Collection of Agricultural and Industrial Microorganisms (NCAIM). All strains were stored in cryobanks.

### 2.3. Preparation of Chicory Root Extract

Fresh chicory roots (*Cichorium intybus* L.) were purchased from a local chicory grower after harvesting and processing including cutting off the lettuce heads (Bakor Ltd., Skierniewice, Poland). The roots were ground to 3 mm size. Initial tests of extraction efficiency and extractability of bioactive ingredients from chicory root were carried out under various conditions and using different solvents, and 4 extracts with the best properties were selected for detailed research. Water extract of chicory root was obtained by pressure boiling at 110 °C for 20 min (pressure vessel 5 L, PS-5682 Vienna, Austria) at a chicory-to-water ratio of 1:3 (*w*/*v*) to obtain water extract (W). The extraction was additionally conducted in the presence of pectinase to enhance the process efficacy, and the water-enzymatic extract (WE) was obtained. For this purpose, the suspension of ground chicory root and water (1:3, *w*/*v*) was adjusted to pH 4 using 0.1 mol/L HCl, and the enzyme solution (Browin Ltd., Lodz, Poland) was added at a concentration of 0.2 mL/kg of root followed by extraction at 40 °C for 6 h. A mixture of water and methanol at 70:30 and 50:50 (*v*/*v*) was alternatively used to prepare extracts at 90 and 80 °C, respectively (WM70/30 and WM50/50). The obtained extracts were cooled in a water bath at 20 °C and filtered under a vacuum pump to separate the extract from the pulp (KNF 18035.3 N, KNF Neuberger, Trenton, NJ, USA). The extracts were then frozen at −40 °C for 24 h and freeze-dried (Delta 1-24 LSC lyophilizer, Martin Christ GmbH, Osterode am Harz, Germany). The freeze-dried extracts were stored at −25 °C until analysis or use in film preparation.

### 2.4. Preparation of Starch Films

Biodegradable starch films were prepared by dissolving 50 g of potato starch and 40 g of glycerol placed in a beaker with 1 L of water (composition of control films). For the modification of starch properties 0.5 g of phytic acid was added. The chicory extracts in an amount of 10, 20, or 50 g were added to the suspension in order to obtain antimicrobial films with extract concentrations of 1, 2, and 5% in relation to the water and 20%, 40%, and 100% in relation to the starch. The mixture was heated with continuous stirring to reach 85 °C providing solubilization and gelatinization of the starch. The resulting starch gel was poured into trays with a diameter of 30 cm. Then the films were dried in an automatic dryer (POL-EKO Aparatura, Wodzisław Śląski, Poland) for 24 h at 50 °C and then finished drying for 1 h at 80 °C. Prior to analysis, the films were stored for at least 1 day at 53 ± 1% relative humidity (RH) and 20 ± 1 °C in desiccators containing saturated magnesium chloride. At least 3 sheets of foil were prepared for each type of film.

### 2.5. Analysis of Properties of Chicory Root Extracts

#### 2.5.1. Analysis of Sesquiterpene Lactone Concentration

The profile of sesquiterpene lactones (SLs) in chicory root extracts was analyzed by ultra-high-performance liquid chromatography with electro-spray ionization and mass spectrometry (UHPLC-ESI-MS) according to D’Antuono et al. [25] with some modifications. The extracts were dissolved in ultrapure water (20 mg/mL) and filtered through a 0.2 μm nylon syringe filter. Chromatographic analysis was carried out using a CBM-20A controller, 2 LC-2020AD pumps, a SIL-30AC autosampler, and a CTO-20AC column oven (Shimadzu, Tokyo, Japan) equipped with a photodiode array detector (SPD-M20A, Shimadzu, Tokyo, Japan), and a mass spectrometer (LCMS-2020, Shimadzu, Tokyo, Japan) with an electrospray ionization source. Chromatographic separation was conducted using an Accucore 150 C18 column (150 mm × 3.0 mm × 2.6 μm; Thermo Scientific, Waltham, MA, USA) kept at 30 °C. The extract solution was placed in the autosampler, 4 μL of sample was injected onto the chromatographic column, and gradient elution was performed. Mobile phase solvent A consisted of water and formic acid (99.9:0.1, *v*/*v*) and solvent B of methanol and formic acid (99.9:0.1, *v*/*v*). A flow rate of 0.2 mL/min was used. The gradient elution was as follows: 0–20 min with 100–58% A, 20–30 min with 58% A, 30–45 min with 58–0% A, and 45–50 min with 0% A. Calibration curves were constructed for standard compounds using 6 concentrations in the range of 0.01–1.0 mg/mL. MS spectra were obtained in collision-induced dissociation (CID) mode using nitrogen. The mass spectrometry conditions were as follows: capillary voltage 4500 V, drying gas temperature 250 °C, drying gas flow 15.0 L/min, nebulizing gas pressure 0.1 MPa, capillary temperature 350 °C, and nitrogen used as the nebulizer. Full-scan mass spectra were acquired over a mass range from *m*/*z* 50 to 2000 in the negative ion mode. The identification of SLs was based on a comparison of retention times, UV–VIS spectra characteristics at 265 nm, and mass spectra with those of authentic standards analyzed under identical conditions, which were characterized with MS ions at [*m*/*z*]^−^ 275 for lactucin and [*m*/*z*]^−^ 409 for lactucopicrin. For instrument control, data acquisition, and evaluation, LabSolutions 5.60 SP2, Chromatography Data System, and Postrun Analysis software, respectively, were used.

#### 2.5.2. Analysis of Reducing Sugars and Nonreducing Carbohydrate Concentration

An analysis of sugars was performed using the Schoorl-Rogenbogen method combined with the Lane-Eynon method with some modifications [26]. To prepare a stock solution, 1 g of the dried extract was dissolved in 50 mL of water; the proteins were removed by precipitation with Carrez reagent, and the content of reducing sugars was determined by reducing Cu^2+^ to Cu^+^. The reaction was performed by titrating boiling Fehling reagent with the reducing sugar solution. In the second part of the analysis, acidic hydrolysis of nonreducing carbohydrates to reducing sugars was performed with the deproteinated stock solution, and the content of reducing sugars was determined again. After analysis, the concentration of nonreducing carbohydrates involving saccharose, fructooligosaccharides with polymerization degree from 3 to 9, and inulin consisting of more fructose units was calculated based on the difference between reducing sugars before and after hydrolysis.

#### 2.5.3. Analysis of Total Polyphenol Content

The content of total phenolic compounds was determined by the use of Folin–Ciocalteu (FC) reagent according to the method described by Sinkowic et al. [27]. The reaction mixture, containing 50 μL of 1% chicory extract solution, 3.85 mL of distilled water, and 100 μL of FC reagent, was prepared, 1 mL of saturated Na_2_CO_3_ solution was added, and the mixture was incubated in the dark at 25 °C for 30 min. After incubation, the absorbance was measured at 725 nm using a T60 (PG Instruments, Lutterworth, UK). The concentration of total phenolic compounds was calculated using a standard curve of chlorogenic acid and expressed as mg of chlorogenic acid equivalent, because this compound is the major polyphenol found in chicory.

#### 2.5.4. Measurement of Color Parameters

The color of the dried extract was measured instrumentally using a CIEL*a*b* Chroma Meter CR-400 colorimeter (Konica Minolta, Osaka, Japan). The color parameters were measured 5 times for each extract. Parameter L* described the brightness of the extract from 0 (black) to 100 (white), while the other parameters describing chromaticity were a* from −50 (green) to +50 (red) and b* from −50 (blue) to +50 (yellow) [28].

#### 2.5.5. Antibacterial Activity of Chicory Root Extracts

The effect of chicory extracts on the growth of selected bacterial strains was investigated using a microculture method (microliter plate assay) modified by Nowak et al. [29]. The bacterial strains were activated in TSB medium with 1% Tween 80 to obtain 10^7^ colony-forming units (CFU)/mL. The lyophilized chicory extracts were dissolved in distilled water (1 g/mL) and added to the media to reach concentrations of 1%, 2%, and 5% (*v*/*v*). The media were inoculated with bacterial strains at 10^4^ CFU/mL. The control samples consisted of bacterial cultures without extracts. The wells of 96 microliter plates were filled with 200 µL of samples and incubated at 30 °C until stationary phase was achieved. Bacterial growth was determined by measuring absorbance at 540 nm using an Asys UVM340 microtiter plate reader (Biogenet, Józefów, Poland). OD540 values were converted to CFU/mL using calibration curves prepared for all types of microorganisms. The bacterial cell number was fitted to the Gompertz equation using an Excel add-in, DMFit 2.1 (Institute of Food Research, Norwich, UK): L(t) = A + C exp{−exp[−B × (t − M)]}. The growth parameters estimated from the equation were maximum specific growth rate µ_max_ = BC/e and lag time tLag = M − (1/B).

### 2.6. Analysis of Film Properties

#### 2.6.1. Water Content Analysis

The water content of the films was determined by the thermogravimetric method at 105 °C for 24 h, after which the drying was prolonged until the mass did not change by more than 0.004 g after subsequent weighing every 30 min [30].

#### 2.6.2. Thickness Measurements

The thickness of the tested films was measured with an electronic digital micrometer (NSK Digitrix Mark II, Japan Micrometer Mfg., Osaka, Japan). Measurements were made in 5 parts of each sheet of foil (1 in the center of the film and 4 around its perimeter) at least in triplicate. For calculations, mean values were taken [31].

#### 2.6.3. Scanning Electron Microscopy Imaging

Scanning electron microscopy imaging was performed with the use of a SEM-JEOL JC M 6000 (Jeol, Tokyo, Japan) at magnification of 200× and 5000×. The following conditions were used: time for coating (spraying) samples with gold, −30 s; accelerating voltage in scanning microscopy, −5 KV; and high-vacuum secondary electron imaging (SEI) picture taking mode [32].

#### 2.6.4. Measurement of Color Parameters

The color of the films was measured similar to the extracts. The color of each film sheet was measured 5 times.

#### 2.6.5. Tensile Strength Measurement

The tensile strength of the films was measured instrumentally by stretching with the EZ Test texturometer (Shimandzu, Kyoto, Japan) using Trapezium software. The breaking force was measured by stretching a 2.5 × 5.5 cm piece of film lengthwise, which was placed in 2 A/TG probe grips, one connected to the table and the other to the movable arm of the apparatus [32]. The initial distance between the grips and the initial velocity were adjusted to 55 mm and 1 mm s^−1^, respectively. Five tensile measurements for each sheet of film were done. The tensile strength was defined as the force registered at complete tearing of the film.

#### 2.6.6. Analysis of Swelling in Water

Swelling of the films in water was measured using a modified Gontard method [31]. Squares of 2 × 2 cm were cut from the film sheets, weighed, immersed in distilled water at 25 °C for 2 min, then thoroughly dried with filter paper to remove excess water and weighed. Immersion and drying were repeated twice more. The amount of water absorbed was calculated as a percentage of the initial mass after 6 min of immersion. Measurements were made after 2 and 4 min to capture possible earlier disintegration of the film.

#### 2.6.7. Water Vapor Permeability Analysis

Water vapor permeability was determined according to the method described by Basiak et al. [30] with some modifications. A sample of 10 g of anhydrous calcium chloride (water vapor pressure 0 Pa) was placed in a round measuring vessel, covered with a *Φ* 70 mm film circle, fixed with a ring, weighed, and placed in a climatic chamber (KBWF 720, BINDER GmbH, Tuttlingen, Germany) at 25 °C and 50% relative humidity (water vapor pressure 1585 Pa). Then, the vessel was weighed every hour for 9 h and the average hourly weight gain, −Δm, was calculated. Water vapor permeability, expressed as the rate of water vapor transmission (WVTR, g/[m^2^*d]), was calculated as the mass (g) of water vapor that permeated through a unit of surface area (m^2^) per day, from the weight gain over time based on the equation: WVTR = Δm × 24/A, where A is the sample active surface area.

#### 2.6.8. Light Transmittance Measurement

Light transmittance measurement was performed using a Luxometer (Voltcraft MS-1300, Hirschau, Germany) [33]. Light intensities at different wavelengths were measured using a barrier in the form of a film between the light source and the light intensity sensor: UVA (340 nm, with initial intensity of 3000 lx), UVB (310 nm, 7200 lx), LED white (483, 595 nm, 8600 lx), and direct day lighting (400–700 nm, 9800 lx). Transmittance was calculated as the percentage of light intensity under the film in relation to that above (initial value).

#### 2.6.9. Antimicrobial Activity

The antimicrobial activity of the films was studied using a modification of Macaremi et al.’s [32] method against Gram-negative (*E. coli*, *P. fluorescens*) and Gram-positive (*S. aureus*, *B. subtilis*) bacteria, and fungi (*C. albicans*, *A. niger*). Bacteria were cultured on TSA medium at 30 °C for 48 h, yeast on Sabouraud medium under the same conditions, and mold on MEA medium at 25 °C for 5 days. Petri dishes containing suitable medium were arranged in 2 parallel rows of inoculum of tested microorganisms with a density of 1–2 × 10^6^ CFU/mL (fungi) or 1–2 × 10^8^ CFU/mL (bacteria) and a length of 8 cm. Then, film strips 2 × 8 cm were placed perpendicular to the growth line and incubated under the conditions stated above. Starch films without the addition of chicory extract were used as controls. After incubation, the zones of growth inhibition under and around the film strips were determined and compared with the scale presented in Table 2.

### 2.7. Statistical Methods

Statistical analysis was based on determining the mean value and standard deviation of at least 5 measurements for each of the 3 sheets of film using Statistica 13.1 software. In order to assess the normal distribution of the groups, the Shapiro–Wilk test was performed. Additionally, Levine’s test was performed to confirm the homogeneity of variance, followed by one-way analysis of variance (ANOVA) to compare the results and Tukey’s test to reveal the pairs of groups that differed with statistical significance in term of the means. Significance was defined at *p* ≤ 0.05.

## 3. Results and Discussion

### 3.1. Chemical Composition and Properties of Chicory Root Extracts

Chicory root, which is a waste product in the food production chain, contains a number of compounds with antimicrobial properties. The main groups of compounds showing antimicrobial properties are polyphenols and sesquiterpene lactones [34]. Sesquiterpene lactones are terpenoid metabolites found in several plant families, most abundantly in *Asteraceae* [35]. The most well-known sesquiterpene lactones in chicory are lactucin, lactucopicrin, 8-deoxylactucin, and 11(S)13-dihydrolactucin [36]. Chicory roots are used on an industrial scale to obtain inulin or, after roasting, to make a coffee substitute. The content of polyphenols, mostly hydroxycinnamic acids, and sesquiterpene lactones in chicory root is 0.2 and 1.0 g/100 g, respectively, on average [37]. The chicory root to be incorporated in the starch-based film must be in the form of an extract added during the starch gel preparation. The extract should be easily soluble in water to avoid the effect of emulsification with the gel. For this reason, extracts from ground chicory root were prepared using water as the solvent to obtain a water extract (W). In the modified variant, pectinase was added during the extraction to improve hydrolysis of the cell wall material and facilitate the extraction of soluble compounds, obtaining a water–enzyme (WE) extract. A mixture of water and methanol in two proportions, 70/30 and 50/50, was also used for extraction, taking into account the possibility of improving the solubility of the antimicrobial active compounds of the chicory root. Water–methanol extracts (WM70/30 and WM50/50) were obtained this way.

The best effects of the extraction of sesquiterpene lactones and polyphenols were obtained using water and water–enzyme extraction. These were, respectively, 4.514 and 5.581 g/100 g dry basis (db) of sesquiterpene lactones and 1.742 and 1.659 g/100 g db of polyphenols in both extracts (Table 3). Our results confirmed the earlier observations of Nwofor et al. [12] and Massound et al. [9] showing that chicory root extract is a very rich source of bioactive compounds with antimicrobial potential. For both sesquiterpene lactones and polyphenols, the use of methanol along with water during extraction resulted in a concentration reduction by about 30% in WM70/30 and an average of 45% in WM50/50. There are many studies available describing the composition of polyphenols in chicory root, establishing that the main group of polyphenols consists of hydroxycinnamic acids and their esters with quinic acid, which are characterized by very good water solubility [38,39].

In addition to polyphenols and sesquiterpene lactones, carbohydrate concentrations in extracts have been determined. These compounds can potentially stimulate bacterial growth [40]. The content of reducing sugars, fructose and glucose increased with increasing methanol content in the extraction mixture, from 11.17 and 12.55 g/100 g db in WE and W to 14.62 and 15.81 g/100 g db in WM 70/30 and WM 50/50, respectively (Table 3). In contrast, the content of nonreducing carbohydrates, consisting mostly of inulin, followed by fructooligosaccharides and saccharose, was reduced from 62.81 and 66.92 g/100 g db in WE and W to 59.42 and 52.69 g/100 g db in WM 70/30 and WM 50/50, respectively. The decreased concentration of nonreducing carbohydrates in extracts obtained with the partial use of methanol, according to Zeaiter et al. [41], was due to the reduced solubility of fructooligosaccharides and inulin in alcohols.

The purpose of the extraction itself was to obtain a preparation that could be obtained as simply as possible, with a composition that was potentially the most beneficial in terms of inhibiting bacterial growth. Considering one-step simple extraction, it was not possible to eliminate carbohydrates, but considering concentrations of bioactive compounds, W and WE extracts had the highest antimicrobial potential.

### 3.2. Antibacterial Activity of Chicory Root Extracts

In order to check whether the extracts had inhibitory effects on microorganisms, a microbiological microplate assay was carried out. The number of microorganisms determined on the basis of OD540 nm measurements was used to calculate the parameters of the curve of bacterial growth. The Gompertz function was adopted for this purpose. After fitting the empirical data to the Gompertz function, a correlation coefficient ranging from 0.9678 to 0.9999 was obtained. Parameters such as the maximum specific growth rate (*µ_max_*) and lag time (*t_Lag_*) were calculated based on the equations obtained. The parameter *μ_max_* is defined as the slope of the tangent line at the inflection point, and *t_Lag_* is the duration of the lag phase (the period between the introduction of a microorganism into a culture medium and the time point at which it begins to increase exponentially).

The chicory root extracts affected the studied strains of bacteria to varying degrees (Table 4 and Table 5). In general, the extracts showed greater antibacterial activity against Gram-negative bacteria, i.e., *E. coli* and *P. fluorescens*. In the case of *E. coli*, WM 70/30 was the most active; at a concentration of 2%, it significantly reduced *µ_max_* by approximately 35%, and at a concentration of 5%, it reduced the value by 70%, compared to the control (Table 4). The W and WM 50/50 extracts were active against this bacterial strain only at a concentration of 5%, reducing *µ_max_* by 50 and 35%, respectively. Lower concentrations led to an increase in *µ_max_* in the range of 15–60%. WE was not active even at the highest concentration used, and it contributed to an increase in *µ_max_* by as much as 25–140%. In the case of the growth parameter *t_Lag_*, it was observed that each extract, irrespective of the concentration, caused its elongation, which increased with increasing concentration of the extract. W and WE at 5% increased *t_Lag_* up to twofold compared to controls. Similar observations were made by Verma et al. [42], who analyzed the effect of water and methanol chicory extracts on *E. coli*, finding better a growth-inhibiting effect of the methanol extract. The mechanism of this difference was not described, but in the work of Nandagopal et al. [43], it can be seen that the water extract contained almost no volatile compounds, which in methanol extract (where they have been detected) may act synergistically with polyphenols and sesquiterpene lactones against the *E. coli* cell wall.

The second Gram-negative strain, *P. fluorescens*, was more sensitive to chicory root extracts. Starting with a concentration of 1%, all extracts reduced *µ_max_* at a comparable level, and WM 70/30 to the greatest extent; at a concentration of 5%, it reduced the exponential growth rate by up to 84% (Table 4). W extract showed similar activity, whereas WM 50/50 and WE were relatively less active, while *tLag*, except for the addition of W at a concentration of 5%, decreased in the presence of extracts.

In the case of Gram-positive *Bacillus subtilis*, the most active were water extracts W and WE, in particular W, which effectively reduced *µ_max_* at a concentration of 1%, and at 5% it completely inhibited the growth. The reduction was similar for WM 70/30, but with the addition of 2% extract, a favorable limitation of the maximum exponential growth rate was observed (Table 4). In contrast, WM50/50 increased *μ_max_* even at a concentration of 5%. Extracts W and WM 70/30 were characterized by a very long *g*, amounting to 497 and 127 h, respectively, and for WM 50/50, the parameter also confirmed the ineffectiveness of this extract in inhibiting the growth of *B. subtilis*, except that the extract in turn contributed to the extension of *t_Lag_* at a concentration of 2%, while others only extended the lag phase at a concentration of 5%, and WM 70/30 in this respect did not work favorably.

Extracts had similar effects on Gram-positive *S. aureus*. In this case, extracts obtained using water limited bacterial growth, in particular W, followed by WE; the *μ_max_* at each concentration of these extracts was lower than that of the controls (*p* > 0.05) (Table 4). The water–methanol extract WM 50/50, however, inhibited its growth rate only at a concentration of 5%, and WM 70/30 was not active in any of the used concentrations. Hence, it is not surprising that only this extract lengthened the *t_Lag_* of *S. aureus* (Table 5).

According to the results, chicory root extracts affected growing bacteria to varying degrees. In general, the influence of extracts on *µ_max_* was observed (Table 4). The most sensitive bacterium was *P. fluorescens*. In this case, all extracts, irrespective of the solvent used, decreased the *µ_max_* value. *S. aureus* was the least sensitive. In the case of this species, only water extracts had a statistically significant effect on this growth parameter. Differences in the sensitivity of Gram-positive and Gram-negative strains may be due to the different mechanism of action of polyphenols and sesquiterpene lactones on the cell wall, attributable to the polar groups of these compounds [44,45]. Additionally, the presence of an α-methylene-γ-lactone group attached to the SL molecule may act as Michael-type acceptor of biological nucleophiles [45]. The lactucin and lactucopicrin and their derivatives present in the tested extracts may be antimicrobial factors due to this mechanism. These compounds were present in the highest concentration in water extracts, which showed the strongest effect on the growth rate of the tested microorganisms (Table 4). The duration of the lag phase increased statistically significantly only in the case of *E. coli* cultures with the addition of all tested extracts regardless of concentration (Table 5).

To the best of our knowledge, the effect of chicory extracts on the dynamics of microbial growth has not been previously tested. Wińska et al. [46] investigated the effect of synthetically obtained bicyclic lactones on the duration of the lag phase of *E. coli*, *S. aureus*, *B. subtilis*, and *P. fluorescens*. The authors found that these compounds prolonged the lag phase. Moreover, in these studies, the most sensitive bacterial strain was *P. fluorescens*, the growth of which was completely inhibited. The data available in the literature come from research on the effect of chicory extracts on the growth of microorganisms carried out by the agar diffusion method. According to Haitao Liu et al. [47], chicory root extract showed activity against *E coli*, *S. aureus*, *Bacillus thuringiensis*, *B. subtilis*, *Salmonella typhi*, *Penicillium* sp., and *Aspergillus* sp. [47]. This was also confirmed by the research of Amer [48], Shaikh et al. [49], and Afzal et al. [50], who found antimicrobial activity of chicory extracts against *S. aureus B. cereus*, *Salmonella Typhimurium*, *E. coli*, *P. aeruginosa*, *C. albicans*, *A. niger*, and *Fusarium solnai.* According to a study by Nandagopal at al. [43], chicory root extracts showed higher inhibitory effects on *B. subtilis*, *S. aureus*, and *S. typhi* than *Micrococcus luteus* and *E. coli* [51]. Eslami et al. [52] and Badakhasann et al. [53] reported that chicory extract had antifungal activity against *Candida glabrata* and *Candida krusei*, and due to its low cost, availability, and proper taste, may be an alternative to nystatin.

### 3.3. Properties of Antimicrobial Biodegradable Starch Film with Chicory Root Extract and Phytic Acid

#### 3.3.1. Physicochemical Properties of Starch Films

Chicory root extracts were used to prepare starch films in order to improve their antimicrobial properties. The concentrations used were 20%, 40%, and 100% in relation to starch and 1%, 2%, and 5%, respectively, of the starch sols obtained before drying, and the concentration in a sol was used in the description. Similar concentrations were used in other biodegradable antimicrobial film formulations using natural extracts [28]. Using a high concentration of chicory extract is a good approach to utilize a significant part of the chicory root, as it is a waste product from chicory lettuce production. All the films were obtained with the addition of phytic acid as a cross-linking agent. The starch films obtained were analyzed for their physical properties.

The water content of the films was determined, which ranged from 4.38 to 8.47 g/100 g. Basiak et al. [30], reporting similar results to the results presented here, showed that the water content in the starch film was about 4 g/100 g (Table 6). The water content of the film decreased with the increased addition of chicory root extract, while the increased methanol content in the solvent mixture used for extraction resulted in increased water content; however, the use of pectinase decreased it. In turn, the addition of phytic acid alone increased the water content. A potentially higher water content in the film can create more favorable conditions for the growth of microorganisms; therefore, the decrease in this parameter along with the increase in the addition of chicory root extracts led to the expected good antibacterial effects of films with higher concentrations of extracts.

**Figure 1:**
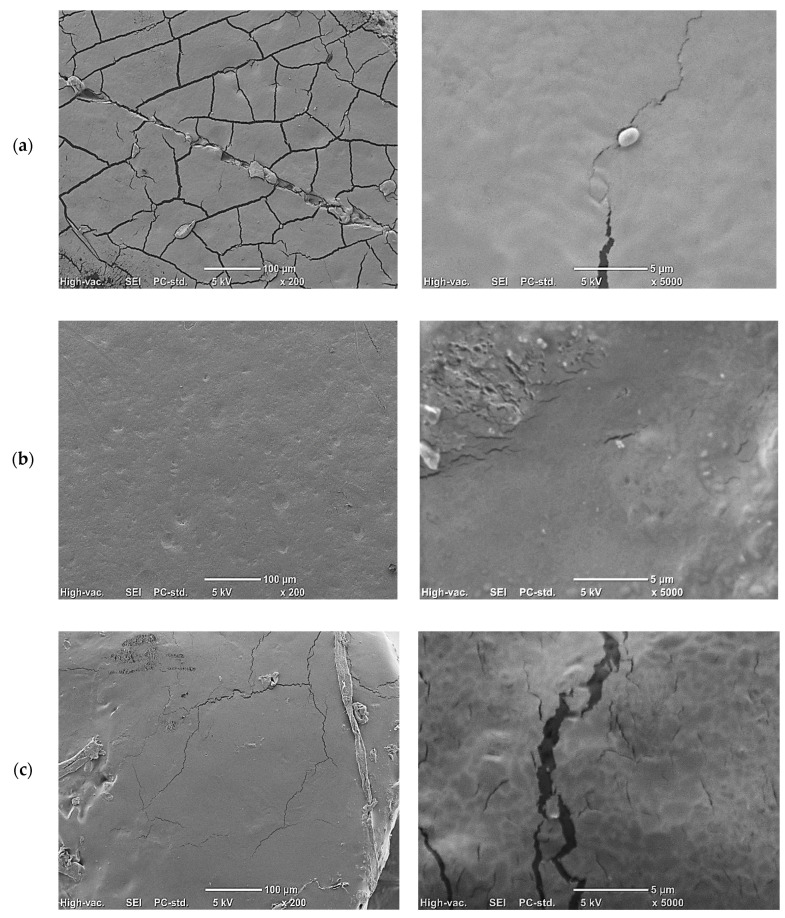

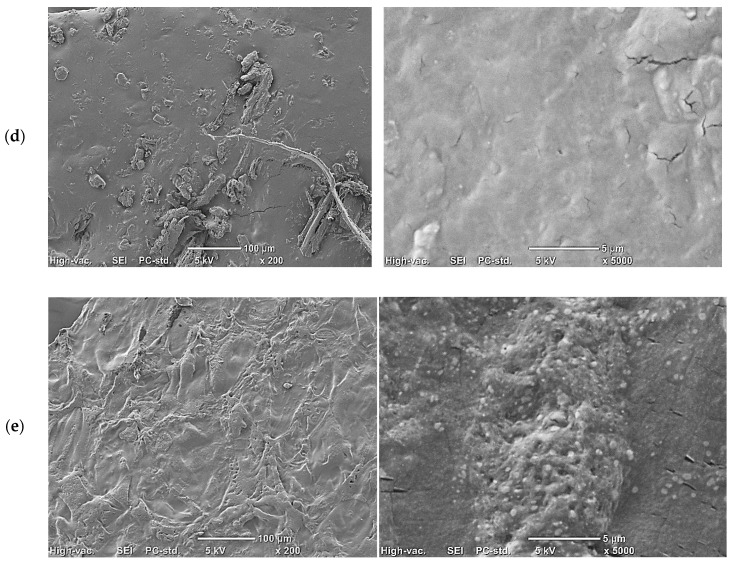
Representative images showing structure of foil using a scanning electron microscope (SEM): (**a**) control, 50 g of starch and 40 g of glycerol dissolved in 1 L of water; (**b**) 50 g of starch, 40 g of glycerol, and 0.5 g of phytic acid dissolved in 1 L of water; (**c**) 50 g of starch, 40 g of glycerol, 0.5 g of phytic acid, and 10 g of chicory root water–enzyme extract dissolved in 1 L of water; (**d**) 50 g of starch, 40 g of glycerol, 0.5 g of phytic acid, and 20 g of chicory root water–enzyme extract dissolved in 1 L of water; (**e**) 50 g of starch, 40 g of glycerol, 0.5 g of phytic acid, and 50 g of chicory root water—enzyme extract dissolved in 1 L of water.

With increased extract content in the films, an increase in their thickness was observed (Table 6, Table S1). The solutions of the films and the increasing concentrations of extracts were characterized by higher viscosity, which resulted in the formation of thicker films when pouring. The control film had a thickness of 0.106 mm, and with phytic acid, it was 0.147 mm. In films with the addition of 5% extract, the thickness increased to a range of 0.160–0.213 mm, while the addition of methanol during extraction reduced the thickness, which could be caused by lower carbohydrate content, especially FW50/50. The differences in thickness were also due to surface folding, as seen in SEM images (Figure 1, Figure S1).

SEM imaging revealed that the control film was characterized by numerous cracks despite the use of a plasticizer in the form of glycerol (Figure 1a). Phytic acid was added to cross-link the starch. This chemical and technological solution was used for the first time to cross-link starch and is a completely new approach to using phosphates in starch processing. Previously, phytic acid was used to protect the starch foil surface, but not to provide modifications of the starch gel [3]. Figure 1b shows that the phytic acid introduced into the film improved the continuity and uniformity of the film structure.

The addition of chicory root extract to the film contributed to the occurrence of aggregates, especially with the 1% extract (Figure 1c), and the folding increased as the concentration of extract increased (Figure 1c–e). This is a typical effect observed with the addition of dried extracts to starch gels, where dispersed extracts form aggregates on the surface after gel drying, contributing to greater film roughness [54]. In the case of the WE extract, a loss of film continuity was observed, with visible cracks, decreasing as the addition of the extract increased (Figure 1c–e).

The tensile strength in the control film was 8.85 N (Table 7). The addition of phytic acid increased the strength to 10.72 N. After adding 1% chicory extract to the film, a reduction in strength to 6.69–8.92 N was observed. The use of methanol during the extraction of chicory root contributed to the reduced mechanical strength of the film. The tensile strength was further reduced by the use of pectinase during water extraction, which was an effect of numerous cracks in the film, especially with 1% WE, visible in the SEM image. Interestingly, increasing the addition of extract resulted in increased tensile strength, to as much as 19.02 N in the case of FW. This drastic increase in the tensile strength was associated, on the one hand, with increased film thickness and folding, and, on the other hand, with greater packing and cross-linking of the structure, as was shown by even lower water content in films containing 5% extract.

Another important feature characterizing the films is their water absorption capacity, described by their swelling susceptibility. This enables the absorption of possible leaks from stored food, leading to greater susceptibility to biodegradation. The swelling was correlated with the content of free water in the films. In terms of swelling, the control film bound the most water. The addition of phytic acid reduced the swelling capacity from 167.34 to 144.59 g/100 g (Table 7). The capacity of swelling was less with water extracts from chicory root added to the films than water–methanol extracts. With 1% extracts, the swelling ranged from 105.45 g/100 g for FWE to 134.06 g/100 g for FWM50/50. As the addition of extracts increased, the capacity of water binding decreased to the range of 82.85–114.24 g/100 g, following the same trend regarding the extract type. This confirmed the binding of starch chains by cross-linking substances, especially phytic acid, where the hydroxyl groups of starch were trapped and unable to bind water.

Water vapor permeability was also tested. This parameter determines the migration of water to and from the outside of the packaging, which in the case of food packaging can be very important for microbial stability and textural features. Water vapor permeability was five times higher for the control film compared to the film cross-linked with phytic acid 4.42 and 16.25 g/(m^2^*d), respectively (Table 7). The addition of chicory root extract to the film caused a further reduction in water vapor permeability. It is worth noting that films with water–methanol extracts added were characterized by lower permeability compared to films with water extracts. In FWMs, smaller thickness was observed, hence greater compression of the structure, which resulted in reduced water vapor permeability. After adding 5% chicory root extract, the water vapor permeability of films was in the range of 6.35–13.24 g/(m^2^*d). Thus, a significant improvement in the barrier properties of the film regarding water vapor permeability was obtained by using the studied additives with cross-linking and antimicrobial properties.

The films were characterized by their color. Brightness L* was the highest for the control film and amounted to 97.82 (Table 8). The film with phytic acid did not differ statistically in brightness. The L* of films with chicory root extracts differed statistically (*p* > 0.05). A correlation was observed between the brightness of the extracts and the films. Those with water extracts, especially those obtained with the use of pectinase, were much darker. With the addition of 5% FWE, L* decreased to 59.74, while the films with other extracts had L* values in the range of 76.69–77.43. Films with chicory root extract were not only darker than the controls but also differed in other color parameters. Within the range of the parameter a*, characterizing the films in terms of the proportion of green and red pigments, the deepest green color, with an a* value of −2.16, was found for the film with phytic acid, while the control film had an a* value of −0.90. The films with chicory root extract added along with phytic acid had a* values in the range of −2.08 to 13.43. The red color deepened with increased additives, and the values for given levels of extracts were similar except for FWE, which showed a far redder color. In the blue to yellow range, only positive b* values were observed, so all films were yellow. The lowest b* value was shown by the control film, at 4.84, while the addition of phytic acid alone caused an increase in b* to 10.54. Extracts from chicory root contributed to an increased b* of the films to the range of 24.95–51.61. In this case, the higher concentration of additive used, the more intense the yellow color. The obtained color parameters of the control film were typical for films based on starch gels [31]. Other studies also showed that the addition of plant extracts significantly deepens the content of yellow and red pigments in films, and this is acceptable to consumers [28].

A very important feature of food packaging is that it limits light transmission, because radiation within a certain wavelength range can contribute to the oxidation of stored food components [33]. In daylight, LED, UVA, and UVB lighting conditions, the films absorbed some of the radiation. While the control film showed light transmission within the mentioned wavelength ranges at a level of 95–98%, the film with phytic acid showed 89–94% transmittance (Table 9). The addition of chicory root extract significantly reduced the light transmission, in particular of UVA. In terms of the type of extract added to the film, the best effect was observed in the case of FWE, then FW, FW70/30, and FW50/50. It is worth noting that in the case of film with 5% chicory water extract obtained with the usage of pectinase, the transmittance of various types of light was in the range of 5–15%. The film was by far the darkest and showed the highest content of red pigments, probably formed as a result of the Maillard reaction, and they could absorb light over a wide range of wavelengths [55]. The extracts were characterized by different color parameters from those of the films; for example, WE was characterized by negative a* (green) and lower b* compared to other extracts. Thus, the Maillard reaction occurred to some extent during the preparation of the films and not only at the stage of freeze-drying the extracts.

#### 3.3.2. Antimicrobial Properties of the Films

Most of the obtained films showed antibacterial activity against both Gram-negative and Gram-positive bacteria. In the case of *E. coli*, the growth-inhibiting activity of most films was good, and in the case of two films, FWM70/30 and FW, with the addition of 1% extracts, low activity was observed (Table 10). Low to very good activity was observed in relation to Gram-positive bacteria. On average, films with water–methanol extracts had better properties. It is worth noting that neither the control film nor the film cross-linked with phytic acid showed antimicrobial activity, although many authors have shown an antimicrobial effect of that substance.

In relation to fungi, the growth-inhibiting activity of films was weaker, in particular against the yeast *C. albicans.* In this case, FW was characterized by low activity regardless of the concentration of the extract in the film. FWM 50/50 and FWE had low activity with higher concentrations of extracts. In relation to mold, the activity of the films was low or good. Similar to the activity against bacteria, both the control film and the film with phytic acid did not show any activity against fungi. Considering the impact of films on the tested microorganisms, it was more beneficial to limit their growth by using films with the addition of 5% extract, in particular water extracts, which were characterized by good activity. In the case of water–methanol extracts, their more compressed structure probably limited the release of active ingredients, as has been observed in the case of fungal growth [54]. The available scientific literature does not contain any studies on the antimicrobial activity of starch films with the addition of chicory extracts or sesquiterpene lactones.

## 4. Conclusions

For the first time, phytic acid was used to improve the physical properties of biodegradable starch film intended for food packaging by cross-linking. The addition of this agent, which intensified the cross-linking of the starch gel, increased the tensile strength of the film, reduced water absorption, and decreased water vapor permeability by a factor of five. The film enriched with phytic acid had no effect on bacterial growth. The film was additionally enriched with extracts of chicory root, which is a waste product in the production of this vegetable. After adding water or water–methanol extracts containing polyphenols and sesquiterpene lactones to the films, their strength increased further, and swelling and water vapor permeability were reduced. The films were visibly darker and contained more yellow and red pigments, which limited the transmission of both visible and UV light. In addition, the films, especially those with water extract, showed a broad-spectrum inhibitory effect on microbial growth. Research has shown the purposefulness of producing biodegradable starch film with both phytic acid and chicory root extract, resulting in packaging that can potentially extend the freshness and microbial stability of stored food; in addition, by using chicory root water extract, the waste of the roots after cutting off the lettuce heads can be significantly reduced. This is a good alternative to using chicory roots for animal feed or as an inulin source or coffee substitute. Further research should be conducted to confirm the bacteriostatic and fungistatic properties of the film when storing various food products.

## Figures and Tables

**Table 1:** Microorganisms used in the study. NCAIM, National Collection of Agricultural and Industrial Microorganisms; ATCC, American Type Culture Collection.

Bacterial Strain	Source	Accession Number
Gram-positive bacteria		
*Bacillus subtilis*	NCAIM	01644
*Staphylococcus aureus*	ATCC	10536
Gram-negative bacteria		
*Pseudomonas fluorescens*	ATCC	13525
*Escherichia coli*	ATCC	10536
Fungi		
*Candida albicans*	ATCC	10231
*Aspergillus niger*	ATCC	16404

**Table 2:** Scale for assessing antimicrobial activity of materials.

Characteristics of Strain Growth	Effect	Symbol of the Effect
Growth inhibition around zone >1 mm and under film	Very good activity	+++
Growth inhibition around zone <1 mm or no growth inhibition around zone and growth inhibition under sample	Good activity	++
No growth inhibition around zone and slight growth inhibition under sample	Low activity	+
No growth inhibition around zone No growth inhibition under sample	Lack of activity	−

**Table 3:** Composition and properties of chicory root extracts.

Composition and Properties	Extract			
	W	WM70/30	WM50/50	WE
Sesquiterpene lactones (g/100 g db)				
8-Deoxylactucin	1.567 ± 0.107 ^a^	1.094 ± 0.084 ^b^	0.809 ± 0.068 ^c^	1.634 ± 0.089 ^a^
Lactucin	0.856 ± 0.072 ^b^	0.554 ± 0.038 ^c^	0.515 ± 0.041 ^c^	2.443 ± 0.170 ^a^
11(S),13-Dihydrolactucin	0.398 ± 0.025 ^a^	0.328 ± 0.025 ^b^	0.285 ± 0.17 ^c^	0.304 ± 0.027 ^b,c^
8-Deoxylactucin oxalate	1.433 ± 0.092 ^a^	1.291 ± 0.074 ^a^	0.946 ± 0.075 ^b^	0.312 ± 0.045 ^c^
Lactucopicrin	0.142 ± 0.008 ^b^	0.141 ± 0.007 ^b^	0.045 ± 0.003 ^c^	0.336 ± 0.030 ^a^
11(Z),13-Dihydrolactucopicrin	0.118 ± 0.005 ^a^	0.083 ± 0.02 ^b^	0.057 ± 0.008 ^c^	0.108 ± 0.008 ^a^
Total sesquiterpene lactones	4.514 ± 0.348 ^b^	3.491 ± 0.208 ^c^	2.657 ± 0.170 ^d^	5.581 ± 0.418 ^a^
Total polyphenols (g/100 g db)	1.742 ± 0.093 ^a^	1.221 ± 0.261 ^b^	1.082 ± 0.105 ^b^	1.659 ± 0.128 ^a^
Carbohydrates (g/100 g db)				
Reducing sugars	12.44 ± 1.04 ^a,b^	14.62 ± 1.32 ^a^	15.81 ± 2.94 ^a^	11.17 ± 1.31 ^b^
Nonreducing carbohydrates	66.92 ± 4.39 ^a^	59.42 ± 3.22 ^b^	52.69 ± 3.28 ^c^	62.81 ± 6.71 ^a,b^
Total carbohydrates	79.36 ± 5.28 ^a^	74.04 ± 4.96 ^a,b^	68.50 ± 5.52 ^b^	73.98 ± 7.04 ^a,b^
Color				
L*	46.11 ± 1.01 ^d^	51.81 ± 1.04 ^b^	56.78 ± 1.18 ^a^	49.21 ± 1.09 ^c^
a*	4.28 ± 0.02 ^b^	4.29 ± 0.01 ^b^	5.38 ± 0.01 ^a^	−1.29 ± 0.01 ^c^
b*	7.21 ± 0.02 ^d^	11.73 ± 0.02 ^c^	16.29 ± 0.03 ^b^	22.11 ± 0.04 ^a^

W, water extract; WM70/30, water–methanol (70/30, *v/v*) extract; WM50/50, water–methanol (50/50, *v*/*v*) extract; WE, water–enzyme extract; L*, a*, b*, color parameters in the CIEL*a*b* system (L*, lightness; a*, redness; b*, yellowness). Same superscript letter in one row indicates no statistically significant differences between extracts (*p* < 0.05).

**Table 4:** Effect of chicory root extracts on maximum specific growth rate (*µ_max_*) of bacteria.

Bacterial Strain	Extract Conc. (%)	Extract			
		W	WM70/30	WM50/50	WE
		*µ_max_* (h^−1^)			
*Escherichia coli*	0	0.204 ± 0.021 ^a^	0.204 ± 0.021 ^a^	0.204 ± 0.021 ^b^	0.204 ± 0.021 ^c^
	1	0.238 ± 0.061 ^a C^	0.200 ± 0.011 ^a C^	0.323 ± 0.097 ^a B^	0.486 ± 0.055 ^a A^
	2	0.231 ± 0.009 ^a B^	0.134 ± 0.011 ^b C^	0.248 ± 0.065 ^a,b A,B^	0.345 ± 0.087 ^a,b A^
	5	0.100 ± 0.042 ^b B,C^	0.064 ± 0.009 ^c C^	0.131 ± 0.025 ^c B^	0.259 ± 0.074 ^b,c A^
*Pseudomonas fluorescens*	0	0.540 ± 0.021 ^a^	0.540 ± 0.021 ^a^	0.540 ± 0.021 ^a^	0.540 ± 0.021 ^a^
	1	0.201 ± 0.007 ^b C^	0.181 ± 0.009 ^b D^	0.511 ± 0.071 ^a A^	0.312 ± 0.007 ^b B^
	2	0.165 ± 0.002 ^c B^	0.117 ± 0.012 ^c C^	0.285 ± 0.069 ^b A^	0.289 ± 0.021 ^b A^
	5	0.106 ± 0.001 ^d B^	0.088 ± 0.007 ^d C^	0.222 ± 0.052 ^b A^	0.281± 0.062 ^b A^
*Bacillus subtilis*	0	0.256 ± 0.052 ^a^	0.256 ± 0.052 ^b^	0.256 ± 0.052 ^d^	0.256 ± 0.052 ^a^
	1	0.114 ± 0.003 ^b D^	0.532 ± 0.023 ^a B^	1.127 ± 0.040 ^a A^	0.282 ± 0.028 ^a C^
	2	0.024 ± 0.006 ^c C^	0.102 ± 0.020 ^c B^	0.639 ± 0.028 ^b A^	0.152 ± 0.064 ^a,b B^
	5	0.001 ± 0.001 ^d D^	0.005 ± 0.001 ^d C^	0.374 ± 0.029 ^c A^	0.136 ± 0.061 ^b B^
*Staphylococcus aureus*	0	0.460 ± 0.022 ^a^	0.460 ± 0.022 ^c^	0.460 ± 0.022 ^b^	0.460 ± 0.022 ^a^
	1	0.117 ± 0.008 ^b D^	0.840 ± 0.009 ^a B^	1.457 ± 0.205 ^a A^	0.292 ± 0.046 ^b C^
	2	0.115 ± 0.012 ^b D^	0.610 ± 0.013 ^b B^	1.360 ± 0.201 ^caA^	0.223 ± 0.021 ^c C^
	5	0.064 ± 0.041 ^c D^	0.464 ± 0.034 ^c A^	0.294 ± 0.090 ^c B^	0.169 ± 0.027 ^d C^

Conc., concentration. W, water extract; WM 70/30, water–methanol (70/30, *v/v*) extract; WM 50/50, water–methanol (50/50, *v/v*) extract; WE, water–enzyme extract. Same superscript letter in one row indicates no statistically significant differences between extracts (*p* < 0.05); lowercase letters indicate statistical significance among different extract concentrations, and capital letters indicate the same concentration of different extracts (*p* < 0.05).

**Table 5:** Effect of chicory root extracts on lag time (*t_Lag_*) of bacteria.

Bacterial Strain	Extract Conc. (%)	Extract			
		W	WM70/30	WM50/50	WE
		*t_Lag_* (h)			
*Escherichia coli*	0	5.5 ± 2.1 ^b^	5.5 ± 2.1 ^b^	5.5 ± 2.1 ^a^	5.5 ± 2.1 ^b^
	1	7.5 ± 3.2 ^a, b A^	8.3 ± 0.5 ^a A^	7.4 ± 3.3 ^a A^	7.5 ± 0.9 ^a,b A^
	2	9.9 ± 1.7 ^a A^	8.9 ± 2.0 ^a,b A^	7.9 ± 1.3 ^a A^	9.6 ± 1.8 ^a A^
	5	10.7 ± 0.8 ^a A^	9.9 ± 2.2 ^a A^	8.7 ± 2.0 ^a A^	10.3 ± 2.2 ^a A^
*Pseudomonas fluorescens*	0	8.6 ± 0.5 ^a^	8.6 ± 0.5 ^a^	8.6 ± 0.5 ^a^	8.6 ± 0.5 ^a^
	1	5.8 ± 0.4 ^b A^	6.5 ± 0.9 ^b A^	5.7 ± 0.2 ^c A^	6.3 ± 0.1 ^c A^
	2	7.3 ± 0.8 ^a A^	6.6 ± 0.3 ^b A^	6.2 ± 0.4 ^b,c A^	6.8 ± 0.4 ^c A^
	5	8.7 ± 1.2 ^a A^	8.0 ± 0.6 ^a A^	6.7 ± 0.2 ^b B^	7.4 ± 0.1 ^b A^
*Bacillus subtilis*	0	9.9 ± 0.2 ^b^	9.9 ± 0.2 ^a^	9.9 ± 0.2 ^c^	9.9 ± 0.2 ^b^
	1	0.2 ± 0.1 ^d C^	0.2 ± 0.1 ^d C^	7.0 ± 0.2 ^d A^	5.9 ± 0.5 ^d B^
	2	5.2 ± 0.1 ^c C^	2.1 ± 0.3 ^c D^	13.5 ± 0.4 ^b A^	8.9 ± 0.1 ^c B^
	5	12.8 ± 0.2 ^a C^	5.7 ± 0.3 ^b D^	26.2 ± 0.9 ^a A^	20.5 ± 0.3 ^a B^
*Staphylococcus aureus*	0	10.0 ± 1.7 ^a^	10.0 ± 1.7 ^a^	10.0 ± 1.7 ^a^	10.0 ± 1.7 ^a^
	1	10.6 ± 3.0 ^a A^	4.8 ± 2.4 ^b B^	4.0 ± 2.7 ^b B^	6.3 ± 2.7 ^a A,B^
	2	10.8 ± 3.2 ^a A^	5.2 ± 2.8 ^b A,B^	2.5 ± 0.1 ^b B^	7.8 ± 4.0 ^a A^
	5	12.8 ± 1.4 ^a A^	6.3 ± 3.3 ^a,b B^	8.3 ± 0.1 ^a B^	8.4 ± 1.4 ^a B^

Conc., concentration; W, water extract; WM 70/30, water–methanol (70/30, *v/v*) extract; WM 50/50, water–methanol (50/50, *v/v*) extract; WE, water–enzyme extract. Same superscript letter in one row indicates no statistically significant differences between extracts (*p* < 0.05); lowercase indicates statistical significance among different extract concentrations, and capital letters indicate the same concentration of different extracts (*p* < 0.05).

**Table 6:** Water content and thickness of starch films with chicory root extracts and phytic acid.

Concentration of the Extract	Film			
	FW	FWM70/30	FWM50/50	FWE
	Water Content (g/100 g)			
Control	7.85 ± 0.52 ^a^	7.85 ± 0.52 ^a^	7.85 ± 0.52 ^a^	7.85 ± 0.52 ^a^
0%	8.03 ± 0.79 ^a^	8.03 ± 0.79 ^a^	8.03 ± 0.79 ^a^	8.03 ± 0.79 ^a^
1%	7.68 ± 0.50 ^a A^	8.03 ± 0.43 ^a A^	8.47 ± 0.39 ^a A^	7.52 ± 0.65 ^a A^
2%	7.29 ± 0.82 ^a A,B^	7.60 ± 0.61 ^a A,B^	8.30 ± 0.84 ^a A^	6.59 ± 0.79 ^a B^
5%	5.25 ± 0.48 ^b C^	6.51 ± 0.22 ^b B^	8.01 ± 0.55 ^a A^	4.83 ± 0.26 ^b C^
	Thickness (mm)			
Control	0.106 ± 0.021 ^c^	0.106 ± 0.021 ^c^	0.106 ± 0.021 ^c^	0.106 ± 0.021 ^c^
0%	0.147 ± 0.018 ^b^	0.147 ± 0.018 ^b^	0.147 ± 0.018 ^a^	0.147 ± 0.018 ^b^
1%	0.150 ± 0.009 ^b A^	0.137 ± 0.012 ^b A,B^	0.135 ± 0.008 ^b,c B^	0.166 ± 0.018 ^b A^
2%	0.171 ± 0.015 ^b A^	0.158 ± 0.011 ^b A^	0.142 ± 0.020 ^a,b B^	0.167 ± 0.020 ^b A^
5%	0.213 ± 0.019 ^a A^	0.200 ± 0.017 ^a A^	0.160 ± 0.011 ^a B^	0.209 ± 0.025 ^a A^

FW, film with water extract; FWM 70/30, film with water–methanol (70/30, *v/v*) extract; FWM 50/50, film with water–methanol (50/50, *v/v*) extract; FWE, film with water–enzyme extract; control, without phytic acid and chicory root extract; 0%, with phytic acid and without chicory root extract. Same superscript letters indicate no statistically significant differences of a given parameter; lowercase indicates statistical significance among different concentrations of the same extract, and capital letters indicate the same concentration of different extracts (*p* < 0.05).

**Table 7:** Tensile strength, swelling, and water vapor permeability of starch films with chicory root extracts and phytic acid.

Concentration of Extract	Film			
	FW	FWM70/30	FWM50/50	FWE
	Tensile strength (N)			
Control	8.85 ± 0.64 ^c^	8.85 ± 0.64 ^c^	8.85 ± 0.64 ^b^	8.85 ± 0.64 ^c^
0%	10.72 ± 0.82 ^b^	10.72 ± 0.82 ^b^	10.72 ± 0.82 ^a^	10.72 ± 0.82 ^b^
1%	8.92 ± 0.47 ^c A^	7.95 ± 0.62 ^c A^	7.38 ± 0.60 ^c B^	6.69 ± 0.57 ^d B^
2%	9.06 ± 0.88 ^c A^	8.62 ± 0.55 ^c A^	7.42 ± 0.39 ^c B^	7.64 ± 0.63 ^c,d B^
5%	19.02 ± 1.14 ^a A^	13.93 ± 0.94 ^a B^	10.79 ± 0.82 ^a C^	15.52 ± 2.12 ^a B^
	Swelling (g/100 g)			
Control	167.34 ± 10.77 ^a^	167.34 ± 10.77 ^a^	167.34 ± 10.77 ^a^	167.34 ± 10.77 ^a^
0%	144.59 ± 9.34 ^b^	144.59 ± 9.34 ^b^	144.59 ± 9.34 ^b^	144.59 ± 9.34 ^b^
1%	123.75 ± 7.15 ^c A^	125.07 ± 6.91 ^c A^	134.06 ± 7.22 ^b A^	105.45 ± 4.58 ^c B^
2%	123.30 ± 5.80 ^c A^	123.08 ± 8.63 ^c A^	131.65 ± 9.38 ^b A^	104.84 ± 6.33 ^c B^
5%	89.22 ± 4.09 ^d C^	99.35 ± 5.60 ^d B^	114.24 ± 3.87 ^c A^	82.85 ± 5.18 ^d C^
	Water Vapor Permeability (G/[M^2^*D])			
Control	74.42 ± 5.28 ^a^	74.42 ± 5.28 ^a^	74.42 ± 5.28 ^a^	74.42 ± 5.28 ^a^
0%	16.25 ± 0.94 ^b^	16.25 ± 0.94 ^b^	16.25 ± 0.94 ^b^	16.25 ± 0.94 ^b^
1%	14.61 ± 0.75 ^b,c A^	13.50 ± 0.82 ^c A^	9.31 ± 0.49 ^c B^	13.46 ± 1.26 ^c A^
2%	13.85 ± 0.92 ^c A^	10.31 ± 0.55 ^d B^	7.66 ± 0.60 ^d C^	11.44 ± 0.93 ^c B^
5%	13.24 ± 1.17 ^c A^	6.83 ± 0.51 ^e C^	6.35 ± 0.64 ^d C^	8.18 ± 0.79 ^d B^

FW, film with water extract; FWM 70/30, film with water–methanol (70/30, *v/v*) extract; FWM 50/50, film with water–methanol (50/50, *v/v*) extract; FWE, film with water–enzyme extract; control, without phytic acid and chicory root extract; 0%, with phytic acid and without chicory root extract. Same superscript letter indicates no statistically significant differences of a given parameter; lowercase letters indicate statistical significance among different concentrations of the same extract, and capital letters indicate the same concentration of different extracts (*p* < 0.05).

**Table 8:** Color of starch films with chicory root extracts and phytic acid in CIELAB color values.

Concentration of Extract	Film			
	FW	FWM70/30	FWM50/50	FWE
	L*			
Control	97.82 ± 0.74 ^a^	97.82 ± 0.74 ^a^	97.82 ± 0.74 ^a^	97.82 ± 0.74 ^a^
0%	96.43 ± 0.52 ^b^	96.43 ± 0.52 ^b^	96.43 ±0.52 ^b^	96.43 ± 0.52 ^b^
1%	89.65 ± 0.37 ^c B^	91.08 ± 0.73 ^cA^	91.51 ± 0.64 ^c A^	83.74 ± 0.73 ^c C^
2%	84.51 ± 0.44 ^d C^	86.07 ± 0.40 ^d B^	87.12 ± 0.55 ^d A^	75.53 ± 0.38 ^d D^
5%	76.69 ± 0.60 ^e A^	77.27 ± 0.27 ^e A^	77.43 ± 0.90 ^e A^	59.74 ± 0.41 ^e B^
	a*			
Control	−0.90 ± 0.03 ^c^	−0.90 ± 0.03 ^c^	−0.90 ± 0.03 ^b^	−0.90 ± 0.03 ^d^
0%	−2.16 ± 0.09 ^e^	−2.16 ± 0.09 ^e^	−2.16 ± 0.09 ^d^	−2.16 ± 0.09 ^e^
1%	−1.49 ± 0.07 ^d B^	−1.91 ± 0.06 ^d C^	−2.08 ± 0.15 ^d C^	0.00 ± 0.01 ^c A^
2%	−0.24 ± 0.05 ^b B^	−0.80 ± 0.01 ^b C^	−1.26 ± 0.06 ^c D^	3.28 ± 0.11 ^b A^
5%	4.73 ± 0.13 ^a B,C^	4.43 ± 0.09 ^a C^	4.90 ± 0.18 ^a B^	13.43 ± 0.16 ^a A^
	b*			
Control	4.84 ± 0.28 ^e^	4.84 ± 0.28 ^e^	4.84 ± 0.28 ^e^	4.84 ± 0.28 ^d^
0%	10.54 ± 0.74 ^d^	10.54 ± 0.74 ^d^	10.54 ± 0.74 ^d^	10.54 ± 0.74 ^c^
1%	24.95 ± 1.20 ^c B^	26.32 ± 2.09 ^c B^	32.22 ± 2,59 ^c A^	23.53 ± 1.61 ^b B^
2%	35.47 ± 1.74 ^b B^	35.81 ± 1.72 ^b B^	38.26 ± 3.14 ^b A,B^	40.34 ± 3.09 ^a A^
5%	48.77 ± 2,83 ^a A^	50.19 ± 2,51 ^a A^	51.61 ± 3.52 ^a A^	42.86 ± 2.94 ^a B^

FW, film with water extract; FWM 70/30, film with water–methanol (70/30, *v/v*) extract; FWM 50/50, film with water–methanol (50/50, *v/v*) extract; FWE, film with water–enzyme extract; control, without phytic acid and chicory root extract; 0%, with phytic acid and without chicory root extract. Same superscript letter indicates no statistically significant differences of a given parameter; lowercase letters indicate statistical significance among different concentrations of the same extract, and capital letters indicate the same concentration of different extracts (*p* < 0.05).

**Table 9:** Light transmittance of starch films with chicory root extracts and phytic acid (%).

Concentration of Extract	Film			
	FW	FWM70/30	FWM50/50	FWE
	Daylight			
Control	95.66 ± 7.38 ^a^	95.66 ± 7.38 ^a^	95.66 ± 7.38 ^a^	95.66 ± 7.38 ^a^
0%	90.56 ± 6.43 ^a^	90.56 ± 6.43 ^a,b^	90.56 ± 6.43 ^a,b^	90.56 ± 6.43 ^a^
1%	75.26 ± 4.27 ^b B^	80.15 ± 6.70 ^b A,B^	86.22 ± 5.02 ^b A^	63.78 ± 2.63 ^b C^
2%	53.52 ± 3.19 ^c C^	62.60 ± 4.73 ^c B^	71.89 ± 3.90 ^c A^	37.65 ± 4.80 ^c D^
5%	50.31 ± 2.98 ^c A^	51.99 ± 2.05 ^d A^	52.96 ± 4.47 ^d A^	13.47 ± 0.54 ^d B^
	LED			
Control	95.81 ± 6.18 ^a^	95.81 ± 6.18 ^a^	95.81 ± 6.18 ^a^	95.81 ± 6.18 ^a^
0%	89.19 ± 5.29 ^a^	89.19 ± 5.29 ^a,b^	89.19 ± 5.29 ^a,b^	89.19 ± 5.29 ^a^
1%	77.67 ± 5.59 ^b A^	77.79 ± 6.58 ^b A^	78.95 ± 7.29 ^b,c A^	62.91 ± 5.18 ^b B^
2%	59.19 ± 3.11 ^c B^	67.91 ± 2.25 ^c A^	70.93 ± 4.30 ^c A^	37.67 ± 3.75 ^c C^
5%	38.72 ± 4.03 ^d B^	50.47 ± 4.06 ^d A^	53.95 ± 4.94 ^d A^	15.47 ± 1.89 ^d C^
		UVA		
Control	96.83 ± 8.15 ^a^	96.83 ± 8.15 ^a^	96.83 ± 8.15 ^a^	96.83 ± 8.15 ^a^
0%	93.67 ± 7.70 ^a^	93.67 ± 7.70 ^a^	93.67 ± 7.70 ^a^	93.67 ± 7.70 ^a^
1%	46.67 ± 3.82 ^b B^	48.83 ± 2.78 ^b A,B^	55.50 ± 4.07 ^b A^	30.00 ± 2.05 ^b C^
2%	22.67 ± 1.90 ^c B^	32.83 ± 2.77 ^c A^	37.00 ± 2.99 ^c A^	15.33 ± 1.27 ^c C^
5%	13.67 ± 0.89 ^d B^	19.50 ± 1.02 ^d A^	21.67 ± 1.28 ^d A^	4.67 ± 0.33 ^d C^
		UVB		
Control	97.92 ± 8.15 ^a^	97.92 ± 8.15 ^a^	97.92 ± 8.15 ^a^	97.92 ± 8.15 ^a^
0%	93.61 ± 7.24 ^a,b^	93.61 ± 7.24 ^a^	93.61 ± 7.24 ^a^	93.61 ± 7.24 ^a^
1%	80.69 ± 7.49 ^b A^	85.00 ± 5.21 ^a A^	88.06 ± 6.00 ^a A^	74.31 ± 5.01 ^b B^
2%	61.25 ± 5.27 ^c B^	65.28 ± 3.94 ^b A,B^	73.06 ± 5.35 ^b A^	34.72 ± 2.77 ^c C^
5%	41.94 ± 3.70 ^d B^	36.25 ± 2.78 ^c B^	55.42 ± 3.98 ^c A^	14.17 ± 1.84 ^d C^

FW, film with water extract; FWM 70/30, film with water–methanol (70/30, *v/v*) extract; FWM 50/50, film with water–methanol (50/50, *v/v*) extract; FWE, film with water–enzyme extract; control, without phytic acid and chicory root extract; 0%, with phytic acid and without chicory root extract. Same superscript letter indicates no statistically significant differences of a given parameter; lowercase letters indicate statistical significance among different concentrations of the same extract, and capital letters indicate the same concentration of different extracts (*p* < 0.05).

**Table 10:** Antimicrobial properties of starch films with chicory root extracts and phytic acid.

Antimicrobial Activity	Film			
	FW	FWM70/30	FWM50/50	FWE
Antibacterial activity				
*Escherichia coli*				
LDPE	–			
0%	–			
1%	++	+	++	++
2%	++	++	++	++
5%	++	++	++	++
*Streptococcus aureus*				
LDPE	–			
0%	–			
1%	+	++	++	++
2%	++	++	++	++
5%	++	++	++	++
*Bacillus subtilis*				
LDPE	–			
0%	–			
1%	+++	++	++	++
2%	++	++	++	+
5%	++	+++	++	++
*Pseudomonas fluorescens*				
LDPE	–			
0%	–			
1%	+	++	++	+
2%	+	++	++	++
5%	++	++	++	++
Antifungal activity				
*Candida albicans*				
LDPE	–			
0%	–			
1%	+	–	–	–
2%	+	–	–	+
5%	+	–	+	+
*Aspergillus niger*				
LDPE	–			
0%	–			
1%	+	+	++	+
2%	+	+	+	+
5%	++	++	++	++

FW, film with water extract; FWM 70/30, film with water–methanol (70/30, *v/v*) extract; FWM 50/50, film with water–methanol (50/50, *v/v*) extract; FWE, film with water–enzyme extract; LDPE low-density polyethylene. For description of the antimicrobial effect, see Table 2.

## Supplementary Materials

The following are available online at https://www.mdpi.com/2304-8158/9/11/1696/s1, Figure S1: Representative images showing the structure of foil using a scanning electron microscope (SEM), Table S1: Thickness spread for one film measured at 5 locations.
