# Supplementary material for: Novel Biodegradable Starch Film for Food Packaging with Antimicrobial Chicory Root Extract and Phytic Acid as a Cross-Linking Agent
Source: Foods. 2020 Nov 19;9(11):1696. doi: 10.3390/foods9111696 (PMC7699324; doi:10.3390/foods9111696)
Supplement: Supplementary file 1 [file foods-09-01696-s001.pdf]

Supplementary materials

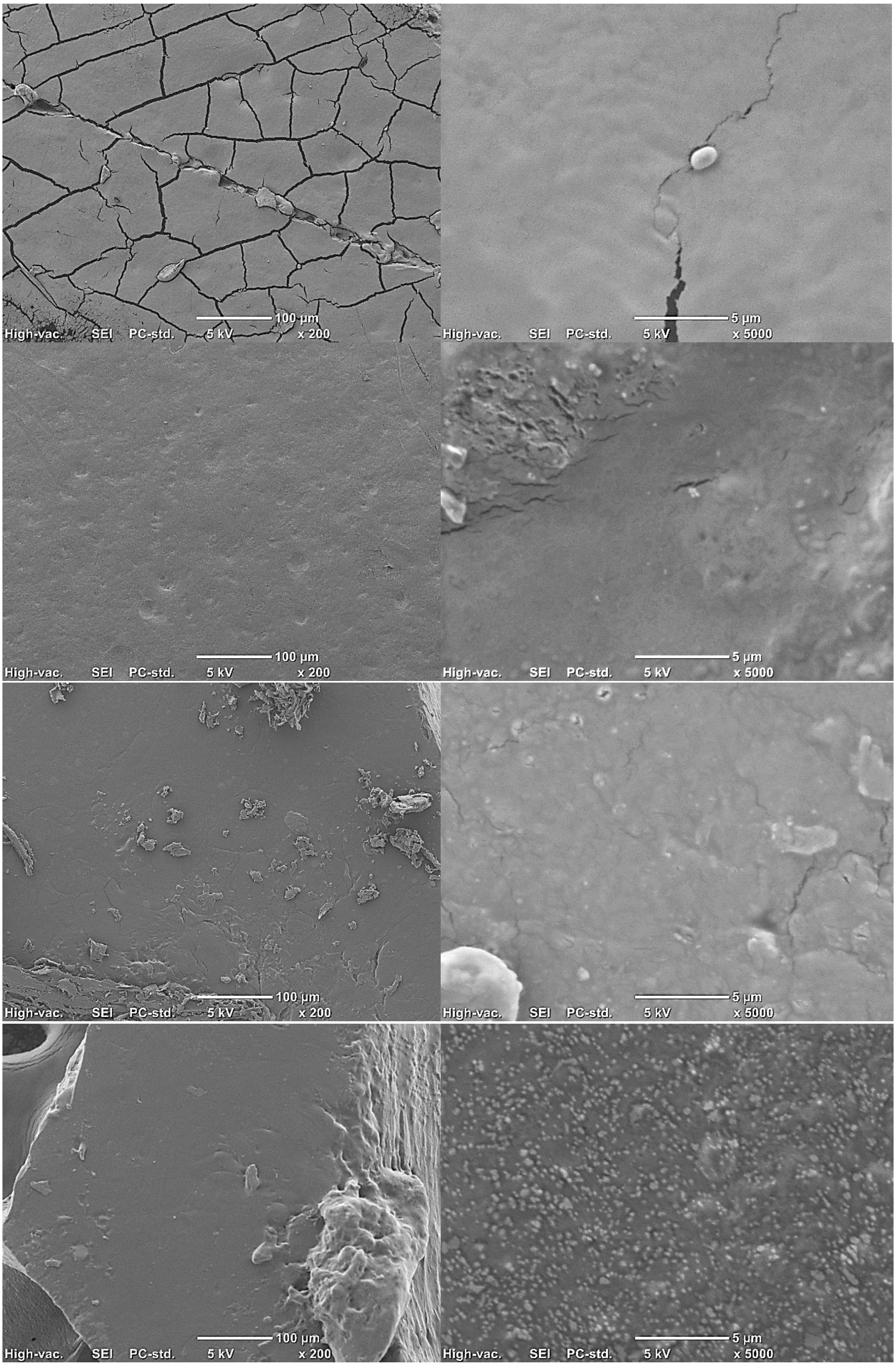

(a)

(b)

(c)

(d)

(e)

(f)

(g)

(h)

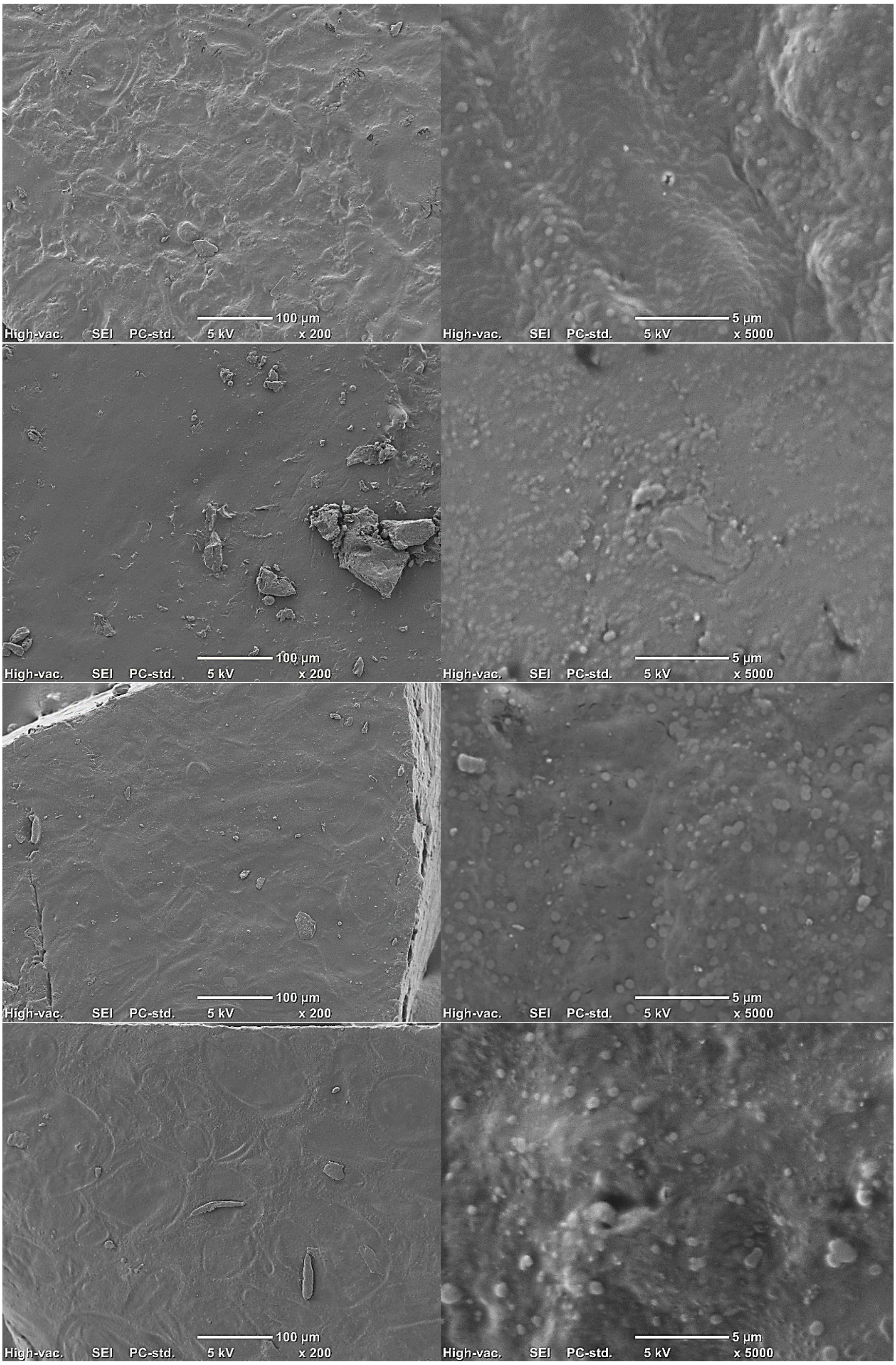
100 µm
High-vac. SEI PC-std. 5 kV x 200
5 µm
High-vac. SEI PC-std. 5 kV x 5000

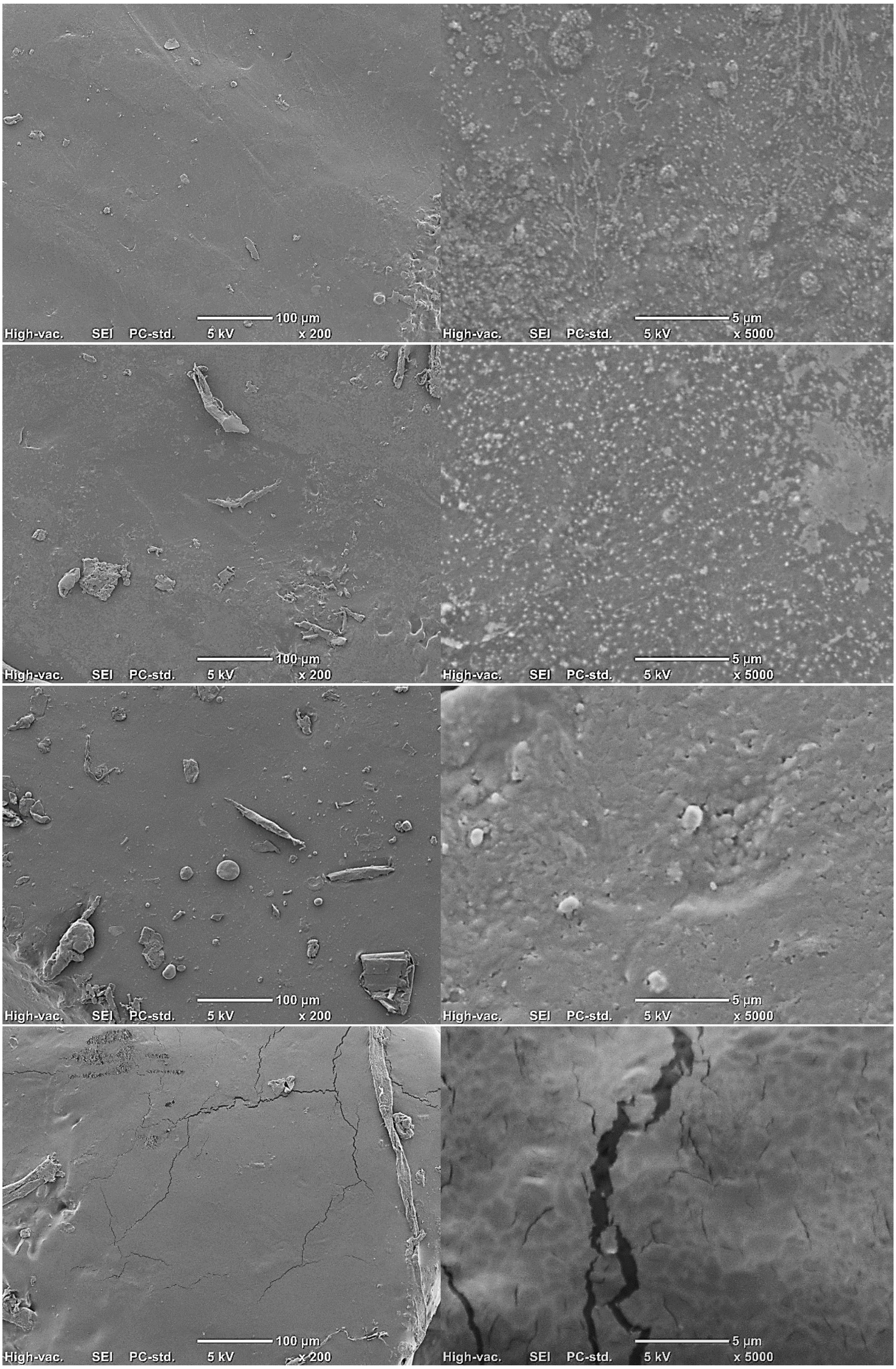
100 µm
High-vac. SEI PC-std. 5 kV x 200
5 µm
High-vac. SEI PC-std. 5 kV x 5000
100 µm
High-vac. SEI PC-std. 5 kV x 200
5 µm
High-vac. SEI PC-std. 5 kV x 5000
100 µm
High-vac. SEI PC-std. 5 kV x 200
5 µm
High-vac. SEI PC-std. 5 kV x 5000
100 µm
High-vac. SEI PC-std. 5 kV x 200
5 µm
High-vac. SEI PC-std. 5 kV x 5000

(i)

(j)

(k)

(l)

(m)

(n)

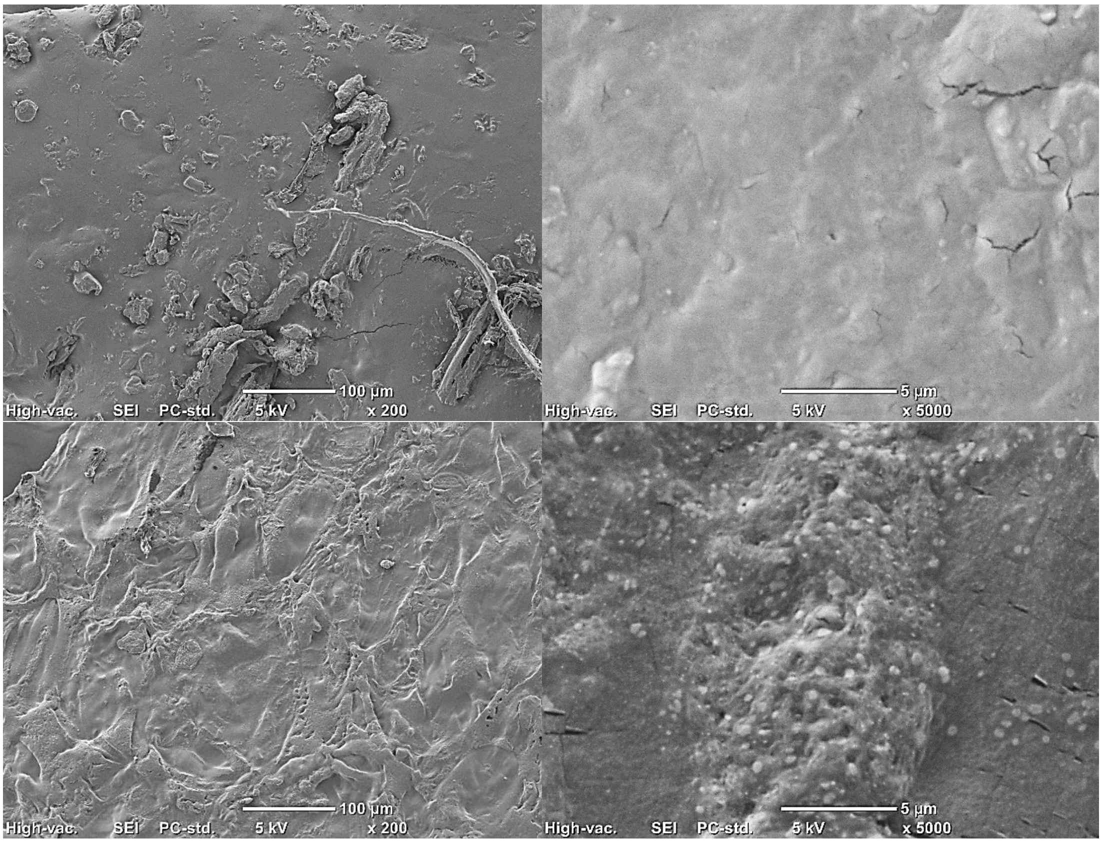

Figure S1. Representative images showing the structure of foil using a scanning electron microscope (SEM): (a) control; (b) with phytic acid; (c) with phytic acid + chicory root water extract 1%; (d) with phytic acid + chicory root water extract 2%; (e) with phytic acid + chicory root water extract 5%; (f) with phytic acid + chicory root water-methanol 70:30 extract 1%; (g) with phytic acid + chicory root water-methanol 70:30 extract 2%; (h) with phytic acid + chicory root water-methanol 70:30 extract 5%; (i) with phytic acid + chicory root water-methanol 50:50 extract 1%; (j) with phytic acid + chicory root water-methanol 50:50 extract 2%;(k) with phytic acid + chicory root water-methanol 50:50 extract 5%; (l) with phytic acid + chicory root water-phytase extract 1%; (m) with phytic acid + chicory root water-phytase extract 2%; (n) with phytic acid + chicory root water-phytase extract 5%.

Table S1. Thickness spread for one film measured at 5 locations

<table>
<tr><th rowspan="3">Concentration of the extract</th><th rowspan="3">Place</th><th colspan="4">Film[1]</th></tr>
<tr><th>FW</th><th>FWM70/30</th><th>FWM50/50</th><th>FWE</th></tr>
<tr><th colspan="4">Thickness (mm)</th></tr>
<tr><td rowspan="7">Control</td><td>1</td><td colspan="4">0.108</td></tr>
<tr><td>2</td><td colspan="4">0.130</td></tr>
<tr><td>3</td><td colspan="4">0.075</td></tr>
<tr><td>4</td><td colspan="4">0.089</td></tr>
<tr><td>5</td><td colspan="4">0.126</td></tr>
<tr><td>mean</td><td colspan="4">0.106</td></tr>
<tr><td>SD</td><td colspan="4">0.021</td></tr>
<tr><td rowspan="7">0%</td><td>1</td><td colspan="4">0.121</td></tr>
<tr><td>2</td><td colspan="4">0.147</td></tr>
<tr><td>3</td><td colspan="4">0.142</td></tr>
<tr><td>4</td><td colspan="4">0.149</td></tr>
<tr><td>5</td><td colspan="4">0.176</td></tr>
<tr><td>mean</td><td colspan="4">0.147</td></tr>
<tr><td>SD</td><td colspan="4">0.018</td></tr>
</table>

1%	1	0.158	0.146	0.139	0.176
	2	0.152	0.141	0.127	0.178
	3	0.155	0.127	0.131	0.129
	4	0.150	0.118	0.129	0.174
	5	0.133	0.151	0.148	0.169
	mean	0.150	0.137	0.135	0.166
	SD	0.009	0.012	0.008	0.018
2%	1	0.183	0.168	0.14	0.171
	2	0.148	0.167	0.179	0.189
	3	0.157	0.152	0.132	0.176
	4	0.183	0.137	0.135	0.166
	5	0.182	0.167	0.122	0.130
	mean	0.171	0.158	0.142	0.167
	SD	0.015	0.011	0.020	0.020
5%	1	0.242	0.198	0.162	0.221
	2	0.201	0.225	0.178	0.249
	3	0.221	0.228	0.148	0.201
	4	0.212	0.189	0.162	0.173
	5	0.187	0.181	0.148	0.199
	mean	0.213	0.200	0.160	0.209
	SD	0.019	0.017	0.011	0.025

[1]FW - film with water extract; FWM70/30 film with water-methanol (70/30, *v/v*) extract; FWM50/50 - film with water-methanol (50/50, *v/v*) extract; FWE – film with water-enzyme extract; control - without phytic acid and chicory root extract.
